# Dr. Joseph R. Woodley

**Published:** 1898-01

**Authors:** W. W. H. Thackston

**Affiliations:** Farmville, Va.


### IN MEMORIUM.—DR. JOSEPH R. WOODLEY.

---

Entered into rest on the 29th day of September, 1897. A distinguished citizen, known and honored among his fellow-men, and among the civil, social, Christian, and professional organizations with which he was associated in his native Southland. A grand and good man has passed away, who was as warmly loved as he was widely known, —Dr. Joseph R. Woodley, of Portsmouth, Virginia, "Has crossed over the River and rests under the shade of the trees."

The annals of the professional and other associations and organizations with which Dr. Woodley was connected are darkened and shadowed by a mortality that has marked a year long to be remembered as a sad and fateful era in the history and records of those fraternal bodies.

The "Grim Reaper" has chosen for his remorseless harvest a startling number of our most honored and esteemed associates and fellow-craftsmen, and of all who have fallen before his ruthless blade none was more beloved, none could be more keenly lamented, none was more highly distinguished for the qualities and attributes that mark and make up the "highest style of man," and we may truly and justly add that none occupied a more enviable position in the profession, which he honored and adorned, than the subject of this memorial.

Socially, Dr. Woodley was known and noted as a great-hearted, brave, and chivalrous gentleman; whose principles were above cavil or criticism, and whose impulses were all general and noble. As a patriot and soldier he bared his bosom to the leaden hail of battle, and from first to last, with the gallant band he led, was always at his post, and between the enemy and the land he loved. Such was Dr. Woodley's skill and dash and daring as a soldier, and so important were the services he performed that he won the recognition and approval of his great captain, Robert E. Lee, expressed in a dispatch, or order, from "Headquarters," through Col. Chas. S. Venable, of his staff, which paper is now in the hands and keeping of the family.

In the varied relations, conditions, and phases of human life it would be difficult, if not impossible, to find a character more symmetrical and well rounded than that of our departed friend.

Professionally, Dr. Woodley measured up to the requirements of the highest positions and offices in the gift of his fellow-craftsmen. Scholarly, skillful, scrupulously and thoroughly faithful in all his work, his gentle ministrations and tender sympathy with his patients won and attached to him a "clientele" perhaps as large as was ever held by a Virginia dentist. In the work of the conventions, congresses, and professional associations, Dr. Woodley was always noted for his ability, enthusiasm, and efficiency; for the broadness of his views and the soundness of his judgment. The Virginia State Dental Association owes its existence and perpetual usefulness largely, if not wholly, to his energy and influence at a critical period in its history, and one of the grandest addresses ever delivered [illegible] dental association was made by Dr. Woodley in the city of Richmond, when he rescued that organization from impending wreck and dissolution. And his contributions have enriched the transactions of the "Southern" and neighboring associations, notably of "North Carolina," to which he was as much devoted as to those of his own native state.

Southern dentists and Southern dentistry have sustained a great and irreparable loss in the death of this noble example of our craft and calling, a bereavement that is shared with the stricken family and wide circle of his sorrowing friends and neighbors, "For none knew him but to love him, none named him but to praise." The great outpouring of the people at his burial and the manifestations of grief and sorrow betokened the high esteem and fond affection in which he was universally held.

Dr. Woodley died, aged sixty-eight years and eight months, and had actively practiced his profession forty-three years. He leaves a widow and three children,—a son and two daughters,—to all of whom we offer our tenderest sympathy and sincere condolence, and for whom we invoke the compassion, the comfort, and the solace of "Him who holds our destinies in the hollow of his hand" and who "tempereth the wind to the shorn lamb." Another "Prince and great man has fallen in our Israel;" we shall see his face and hear, his voice no more, but in our hearts his character, his example, and his memory will be cherished and embalmed while we live to worship truth, to honor virtue, and to find in noble manhood an inspiration and a joy.—W. W. H. THACKSTON Farmville, Va.—*Dental Cosmos.*

